# Cross‐sectional association between blood cholesterol and calcium levels in genetically diverse strains of mice

**DOI:** 10.1002/2211-5463.13757

**Published:** 2024-01-07

**Authors:** Cody M. Cousineau, Kaelin Loftus, Gary A. Churchill, Dave Bridges

**Affiliations:** ^1^ Department of Nutritional Sciences University of Michigan School of Public Health Ann Arbor MI USA; ^2^ The Jackson Laboratory Bar Harbor ME USA

**Keywords:** calcium, cholesterol, cross‐sectional, diversity outbred

## Abstract

Genetically diverse outbred mice allow for the study of genetic variation in the context of high dietary and environmental control. Using a machine learning approach, we investigated clinical and morphometric factors that associate with serum cholesterol levels in 840 genetically unique Diversity Outbred mice of both sexes (*n* = 417 male and 423 female), and on both a control chow (% kcals in diet: protein 22%, carbohydrate 62%, fat 16%, no cholesterol) and high fat high sucrose (% kcals in diet: protein 15%, carbohydrate 41%, fat 45%, 0.05% cholesterol). We find expected elevations of cholesterol in male mice, as well as in mice with elevated serum triglycerides and/or fed a high fat high sucrose diet. The third strongest predictor was serum calcium which correlated with serum cholesterol across both diets and sexes (*r* = 0.39–0.48) in both Diversity Outbred (*P* = 3.0 × 10^−43^) and BXD (*P* = 0.005) mice. This is in‐line with several human cohort studies which show associations between calcium and cholesterol, and calcium as an independent predictor of cardiovascular events.

## Graphical abstract

We used a machine learning approach using data from genetically diverse mice to identify predictors of serum cholesterol in mice. We reproduced well‐established associations between diet and triglycerides on cholesterol levels, but identified a new diet‐independent connection between serum calcium and cholesterol in Diversity Outbred mice.

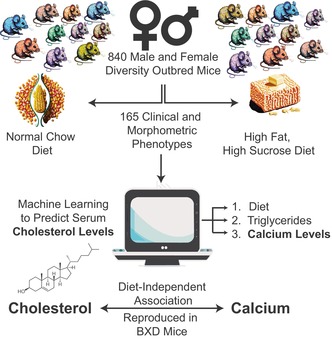

**Abbreviations**
BMI: body mass index
BW: body weight
BXD: B6‐by‐D2
CETP: cholesteryl ester transfer protein
DO: diversity outbred
HDL: high‐density lipoprotein
HFHS: high fat, high sucrose (diet)
LDL: low‐density lipoprotein
NCD: normal chow diet
TG: triglycerides

Video

Elevated blood cholesterol, particularly in the forms of atherogenic LDL particles are causal of cardiovascular disease, the major cause of death in Western societies [1]. Cholesterol levels in humans vary widely depending on multiple factors including genetics, diet and other lifestyle factors, with genetics and lifestyle each contributing roughly equally to cardiovascular disease risk [2].

Human genetics has led to a sophisticated understanding of how cholesterol synthesis and excretion is regulated, and how this varies between individuals and diets [3]. Understanding complex genetic traits like cholesterol is a challenge in human studies where diet and lifestyle data is subject to substantial instrument error. Here genetically diverse panels of mice, where the diet and environment are tightly controlled, and genetics can be well defined, can provide solutions. Among several resources, the Diversity Outbred [4] and UM‐HET3 [5] mice represent genetically diverse outbred populations, while the Collaborative Cross [6] and BXD [7] resources provide data on recombinant inbred mice. Collectively these resources are valuable tools to understand complex traits such as cholesterol.

In this study, we performed a secondary data analysis of Diversity Outbred mice to understand clinical and phenotypic correlates of elevated cholesterol levels. Using a machine learning approach, we demonstrate that sex, diet, and serum triglycerides are strong predictors of cholesterol levels in these mice, but also that even with high dietary control serum calcium provides and independent correlate of serum cholesterol levels.

## Materials and methods

### Diversity outbred data

The phenotype data for diversity outbred mice (RRID:IMSR_JAX:009376) contains data on 840 mice from the diversity outbred collection of both sexes [8]. The dataset includes 162 phenotypes for each mouse, measured once, twice, or weekly in the case of body weights. Animals were first received at wean age (3 weeks old) and then distributed into cages of five same‐sex animals per cage. Mice were housed five same‐sex animals per pen in individually ventilated cages (Thoren #11 Duple II, Thoren Caging Systems, Hazelton, PA, USA) with pine bedding and free access to food and acidified water. Light and dark cycles were 12 h each, with the light cycle starting at 6 AM. Animals were housed in pressurized, individually ventilated cages (Thoren Caging Systems) with pine bedding (Crobb Box, Ellsworth, ME, USA) and had *ad libitum* access to food. At time of weaning, mice were placed on a high‐fat high‐sucrose diet (HFHS; Harlan TD.08811), or kept on a normal chow diet (NCD; LabDiet 5K52). In the final dataset, there were 225 female mice on NCD, 224 male mice on NCD, 198 female mice on HFHS, and 193 male mice on HFHS. Blood from mice was obtained from the retro‐orbital sinus after administration of tetracaine HCl using a heparin‐coated microcapillary tube and collected into a 1.5 mL Eppendorf tube. For collection of blood plasma, approximately 150 μL of whole blood was collected into a tube and plasma was separated by centrifugation at 8000 ***g*** for 10 min at 4 °C and removed into a clean Eppendorf tube. Cholesterol, triglycerides, and calcium were quantified in plasma using the Beckman Synchron DXC600Pro Clinical chemistry analyzer. Body composition was collected by dual x‐ray absorbitrometry on Lunar PIXImus densitometer (GE Medical Systems, Waukesha, WI, USA). All procedures were reviewed and approved by Jackson Laboratory Animal Care and Use Committee (ACUC) under animal summary #06006. Additional details about these mice can be found in [9].

### BXD data

Calcium and cholesterol levels from male and female BXD mice were described in [10]. These data were downloaded from genenetwork (http://www.genenetwork.org/) [11, 12] as datasets BXD_12844, BXD_12914, BXD_12951, and BXD_12881. These datasets included 17 female strains (72 mice) and 36 male strains (254 mice; RRID:MGI:2164899). These mice were maintained on a chow diet (SAFE; D04) with blood collected at 14 weeks of age. Data were averaged and analyzed by strain and sex.

### Statistics

All statistical analyses were performed using r version 4.2.0 [13]. Cholesterol data were not normally distributed within groups (*P* < 0.05 by sex and diet stratified Shapiro–Wilk tests), so non‐parametric pairwise tests were used. Summarized data are reported as mean ± standard deviation for summary statistics and standard deviation of the error for multivariate models. For all comparisons, sex was first tested as a modifier and then as a covariate. If there was significant evidence of sex modification, pairwise sex‐stratified analyses are also reported. Regression trees were generated using the rpart package (version 4.1.19; [14]) and pruned based on the number of branches at the minimum cross‐validated standard error rate. Partial effect sizes were estimated using the effectsize package (version, reporting $\omega_{p}^{2}$ [15, 16]) Causal mediation analyses were performed with 1000 bootstraps with both the full and mediator models adjusted for sex using the mediation package (version 4.5.0; [17]), using the method described in [18]. Statistical significance was set at an alpha of 0.05.

## Results

### Diversity outbred mice exhibit diet and sex dependent variation in cholesterol levels

**Fig. 1:**
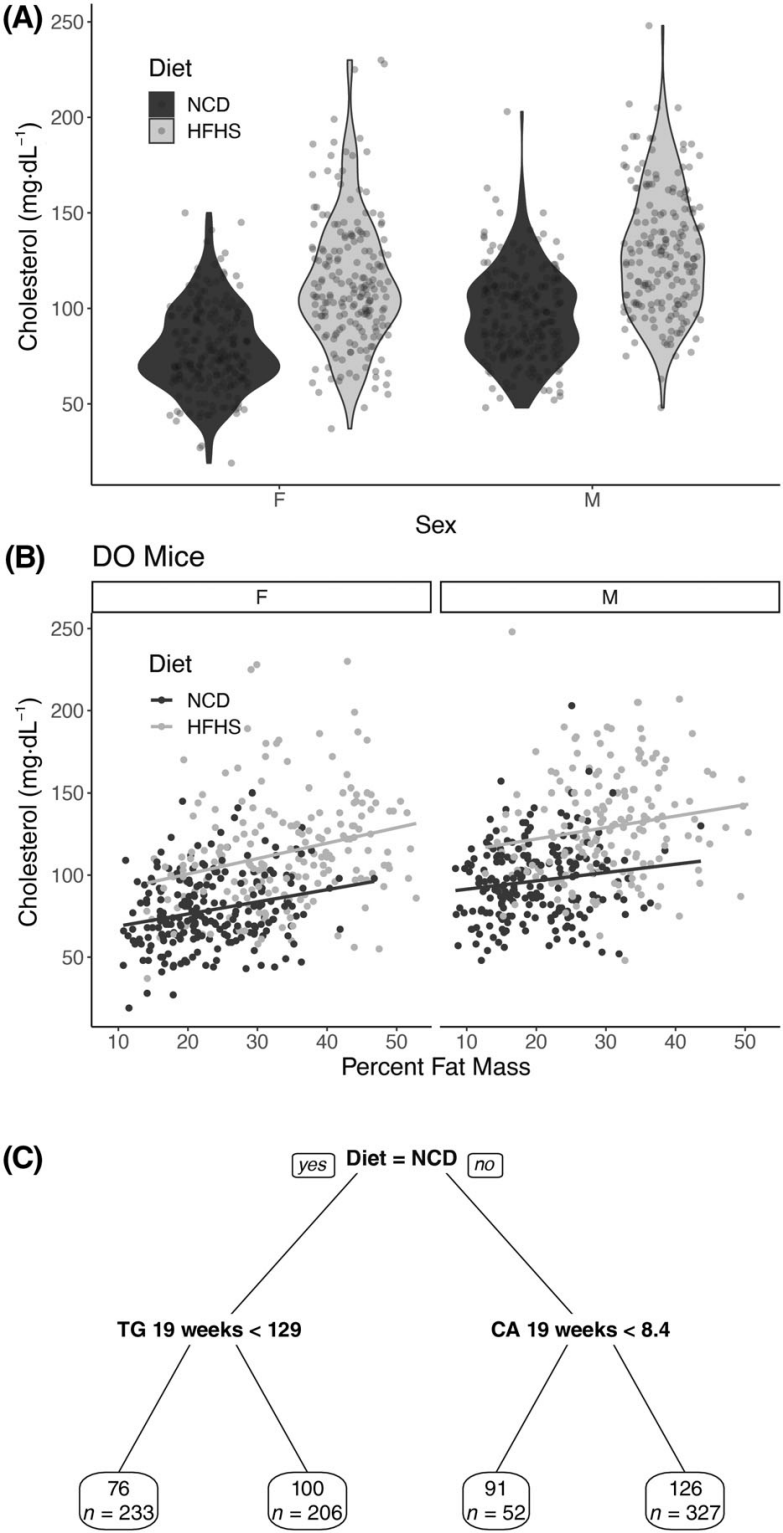
Description of cholesterol levels in diversity outbred mice. (A) Violin plot of cholesterol levels of diversity outbred at 19 weeks, mice stratified by diet and sex. (B) Scatter plot of cholesterol levels in relation to percent fat mass at 19 weeks of age, stratified by diet and sex. (C) Pruned regression predicting cholesterol at 19 weeks. Above the box is the algorithmically generated cutoff for triglycerides (abbreviated TG in mg·dL^−1^), calcium (Ca in mg·dL^−1^), and body weight (BW in g). Each predictor was a phenotype measured at 19 weeks. Within each box, the value represents the average cholesterol level in that group (in mg·dL^−1^) and the number of mice in that group (*n* = 840 for panel A and 818 for panel 2 as 22 mice were omitted due to missing data).

We first evaluated the cholesterol levels in the diversity outbred mice measured at 8 and 19 weeks (5 and 16 weeks of HFHS or NCD feeding). Cholesterol levels for each group were similar at both time points (*P* = 0.474 by pairwise Wilcoxon test, see Fig. S1). This indicates that cholesterol levels are stable between both time points. We stratified cholesterol levels by sex and diet. Via multivariate regression, we found the expected cholesterol elevations in mice on a HFHS diet (33.7 ± 2.0 mg·dL^−1^, *P* = 1.4 × 10^−56^), and male sex (16.9 ± 2.0 mg·dL^−1^, *P* = 3.0 × 10^−17^; Fig. 1A). There was no evidence of a significant interaction between diet and sex (*P* = 0.636).

The HFHS diet is deployed primarily as an obesogenic diet in rodents. We evaluated the role of body weight and fat mass on cholesterol levels in these mice. As expected, after adjusting for sex, increases in both body weight (16.9 ± 1.6 mg·dL^−1^ per 10 g weight; *P* = 2.2 × 10^−24^) and percent fat mass (15.2 ± 1.1 mg·dL^−1^ per 10% increase in percent fat mass; *P* = 3.1 × 10^−38^) correlated with cholesterol levels at 19 weeks of age (Fig. 1B). We next asked whether diet played a role aside from increasing percent fat mass and found that this is true as well. We performed a causal mediation analysis to interrogate this relationship and estimate that, after adjusting for sex, differences in percent fat mass explained 25 percent of the HFHS diet effect on cholesterol levels in these mice (95% CI 17–34%; *P*_mediation_ < 1 × 10^−15^). This suggests that there are both obesity‐dependent and HFHS‐diet‐dependent contributions to cholesterol levels in these mice.

### Diet, triglycerides, and calcium associate with cholesterol levels

To define other potential associations between cholesterol and measured phenotypes in this dataset, we generated a regression tree using the 165 phenotypes in this dataset (Fig. 1C). The major classifier of cholesterol levels was the diet, and the second was triglycerides measured at 19 weeks. Serum calcium measured at 19 weeks was the third phenotype that associated with cholesterol levels, and body weight measured at 19 weeks was the fourth (Fig. 1C).

**Fig. 2:**
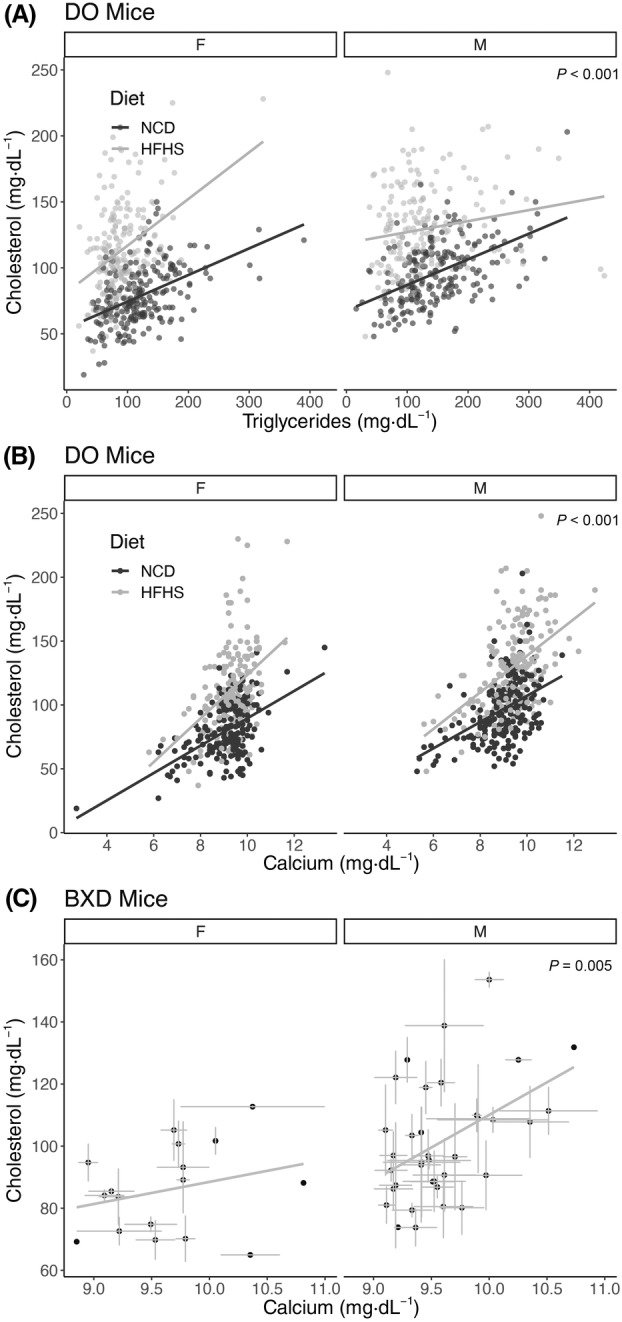
Cross‐sectional associations of cholesterol with triglycerides and calcium. Sex and diet stratified scatter plots of (A) triglyceride and (B) calcium relationships with cholesterol levels at 19 weeks in diversity outbred mice (*n* = 840 mice and strains). (C) Cholesterol and calcium associations in male and female BXD strains (*n* = 326 mice from 17 female and 36 male strains; error bars represent within‐strain standard error of the mean). *P*‐values indicate the level of significance for the diet and sex adjusted relationship between cholesterol and triglycerides or calcium from a multivariate linear model. The lines represent independent estimates for each group.

Dyslipidemia often includes elevations of both triglyceride and cholesterol levels in both mice and humans, so the association of triglycerides with cholesterol was not unexpected. Via multivariate modeling accounting for the effects of diet and sex, a 100 mg·dL^−1^ increase in triglycerides was associated with a 17.7 ± 1.7 mg·dL^−1^ elevation in cholesterol (*P* = 3.8 × 10^−24^, Fig. 2A). Within subgroups, the triglyceride–cholesterol relationship varied somewhat, with weaker correlations in HFHS populations (Spearman's ρ for males 0.190, females 0.276) than on NCD (males 0.491, females 0.453). Via multivariate regression with interactions, we observed significant sex × diet × triglyceride effect modification with respect to cholesterol levels (*P* = 4.5 × 10^–4^, $\omega_{p}^{2}$ = 0.014).

The strong cross‐sectional association of calcium with cholesterol was not predicted by our research team. As shown in Fig. 2B, after adjusting for diet and sex, a 1 mg·dL^−1^ increase in calcium is associated with a 12.7 ± 0.8 mg·dL^−1^ increase in cholesterol (*P* = 3.0 × 10^−43^). Linear models predicting serum cholesterol including sex and diet, and calcium as covariates had an adjusted *R* ^2^ of 0.45 with a partial effect size ($\omega_{p}^{2}$) of 0.22 for serum calcium. We performed sub‐group analyses and found that each diet‐sex combination had broadly similar estimates for Spearman's ρ (ranging from 0.39 for HFHS females to 0.48 for HFHS males), each of which had a *P*‐value of less than 2.2 × 10^−7^. In this study, calcium levels were not affected by rodent sex (*P* = 0.59) and only modestly by diet (increased by 0.3 mg·dL^−1^ in HFHS; Fig. S2A). Due to the strong impacts of diet and sex on cholesterol levels, these data suggest that calcium may work largely independently of those factors.

Given the differences in the physiology and cardiovascular disease associations between HDL versus LDL cholesterol concentrations, we repeated this analysis assessing the diet‐ and sex‐adjusted associations between HDL cholesterol and non‐HDL cholesterol and calcium (Fig. S2B,C). Both of these fractions have positive associations with calcium levels, indicating that the positive associations with calcium are found in both apolipoprotein fractions. The relationships between all clinical parameters and endpoint cholesterol both in terms of non‐parametric correlations and diet/sex adjusted associations are presented in Table S1.

To externally test these findings, we evaluated a distinct dataset of genetically diverse mice, the BXD mouse collection. In a secondary data analysis using data in [10], we replicated this finding, showing a cross‐sectional association between cholesterol and calcium levels after adjusting for sex differences. Similar to the data from the diversity outbred mice, we estimate a 14.8 ± 5.1 mg·dL^−1^ increase in cholesterol was observed for every 1 mg·dL^−1^ increase in calcium (*P* = 0.005). In the smaller BXD dataset, there appeared to be a stronger relationship in male mice (Spearman's ρ = 0.393; *P* = 0.018, *n* = 36 strains) than female mice (ρ = 0.269; *P* = 0.297, *n* = 17 strains), but in multivariate modeling there was no significant modifying effect of sex (*P* = 0.178), potentially due to the relatively small number of female strains.

In the diversity outbred mice, serum calcium levels are not significantly altered by sex (*P* = 0.59), and only modestly increased by HFHS diets (0.30 ± 0.07 mg·dL^−1^; *P* = 5.1 × 10^−5^; Fig. S2C). Since calcium is normally tightly controlled by homeostatic mechanisms regulating calcium absorption and bone remodeling, we tested whether bone mineral content and density in these mice was associated with cholesterol levels. As shown in Fig. S2D,E, there was no evidence of an association between bone mass or density measured at 21 weeks and cholesterol levels measured at 19 weeks (*P* = 0.93 and 0.90, respectively) in the diversity outbred dataset.

## Discussion

In this study, we report an association between calcium and cholesterol in two distinct mouse datasets. This relationship was similar across both sexes and over both normal chow and obesogenic high fat, high sucrose diets. We were not surprised that HFHS feeding raised cholesterol, as this has been widely observed in mice, rats, and humans. This is likely due to a combination of increased dietary cholesterol, triglycerides, and body fat in these mice. The calcium relationship with cholesterol that was identified here is a unique observation in mice. As the magnitude of elevations of cholesterol and calcium from HFHS diet were similar, and because diet did not substantially alter calcium levels, it is possible that calcium and diet are independent predictors of cholesterol homeostasis. The finding that the calcium–cholesterol association is largely independent of sex, diets or triglyceride levels suggests that serum calcium may represent a novel biomarker of dysfunctional cholesterol homeostasis and may point to novel mechanisms by which cholesterol could be controlled.

To our knowledge, this is the first demonstration of an association between serum calcium and cholesterol in rodents. That being said, several large cross‐sectional studies have consistently demonstrated a correlation between serum calcium and cholesterol in multiple populations [19, 20, 21, 22, 23, 24, 25, 26, 27, 28, 29, 30, 31, 32]. In addition, calcium is also a longitudinal predictor of cardiovascular events in humans independent of BMI or blood pressure [21, 32, 33, 34, 35, 36, 37]. A meta‐analysis of these associations show that an increase of one standard deviation of serum calcium is associated with an 8% increased risk of subsequent cardiovascular events [38]. These data are consistent with the hypothesis that the calcium‐cholesterol relationship we report here in mice is concordant with increased cardiovascular risk.

The present study does not speak to the directionality of this association, but there are some hints in the literature. A meta‐analysis demonstrates a 31% increased risk of myocardial infarction in patients supplementing with calcium compared to placebo [39]. Patients with primary hyperparathyroidism have elevated parathyroid hormone and calcium levels and are an interesting population to examine. The results of case–control studies evaluating cholesterol levels are mixed and there is limited evidence that PTH causes elevated cholesterol levels. While reports show that these patients also have significantly elevated total and/or LDL‐cholesterol [40, 41], though most others show either non‐significant effect or even decreases [42, 43, 44, 45, 46, 47, 48].

In terms of whether cholesterol could be driving hypercalcemia, there is some evidence. Two interventional studies using statins have shown reductions in calcium levels [49, 50], whereas another single‐arm study showed declines that did not reach significance [51]. A Mendelian Randomization approach using LDL‐C as the instrument was also associated with elevated calcium levels [52], further supporting a potential causal relationship with cholesterol driving calcium levels. As this is a cross‐sectional associative relationship, we are not able at present to define directionality, much less the underlying biological mechanism(s). Whether calcium can modify serum cholesterol, or cholesterol can modify calcium are both important nutritional and pathophysiological questions, and future controlled mouse studies should shed light on the directions and mechanisms explaining this association.

There are several other strengths of this study. We present data on a large number of mice roughly equally divided between sexes and two diets and find consistent results across all groups. We have exceptional control of confounders such as diets, environment, activity levels, and other exposures that could affect the interpretation of the human studies. Our supervised machine learning approach used a large number of measured phenotypes to predict calcium levels, and set cutoffs in a data‐driven manner. We consider the support of data from two independent genetically diverse mice populations another strength of this work. Calcium–cholesterol associations appear to be robust over a wide variation in genetics and not restricted to findings in inbred mouse populations. As such, this relationship holds over multiple diets, sexes, investigators, sites, and genetic backgrounds.

### Limitations of the present study

While there were multiple measurements of calcium and cholesterol in this dataset (at week 8 and week 19, after 5 and 16 weeks of HFHS/NCD respectively), cholesterol levels were stable at these points. Therefore, it was possible to effectively evaluate the longitudinal association between cholesterol and calcium, nor the effects of advanced age in modifying this relationship. In addition, this cross‐sectional association does not ascribe a directionality to this relationship, at this stage we think it plausible that calcium may increase cholesterol, that cholesterol might increase calcium, or that a third, unmeasured factor drives both factors. As this is a secondary data analysis, we are unable to evaluate differences in calcium‐regulatory hormones, which we predict would vary more than the relatively homeostatic blood calcium levels. Another limitation is that cholesterol homeostasis is substantially different in mice and humans, especially in the fraction of cholesterol present in the HDL versus LDL fractions, due to the absence of CETP in mice. These data therefore largely reflect associations between calcium and the HDL pool. Finally, as cardiovascular disease is extremely rare in mice of this age, we did not assess cardiovascular disease, or atherogenic lesions as an endpoint in this study.

In conclusion, in this work, we use a machine learning approach to describe that diet, sex, triglyceride levels, and calcium all contribute independently to the serum cholesterol levels in Diversity Outbred mice, and that the relationship between calcium and cholesterol holds true in BXD mice. These data support that the observed human relationships between serum and cholesterol levels are true in mice, and present an opportunity for further physiological and genetic dissection of this relationship.

## Conflict of interest

The authors declare no conflict of interest.

### Peer review

The peer review history for this article is available at https://www.webofscience.com/api/gateway/wos/peer‐review/10.1002/2211‐5463.13757.

## Author contributions

DB and CMC conceptualized this research study, decided and validated the methodologies, performed the investigations, wrote the original draft, the final draft, and prepared visualizations. Formal analyses were done by CMC, DB and KL. Data were provided by GAC. This work was administered and supervised by DB who along with GAC performed the data validation. Funding for this work was acquired by DB and GAC. All authors have read and agreed to the final published work.

## Supporting information


**Fig. S1.** Cholesterol levels are stable across time in diversity outbred mice. Average cholesterol levels, and levels measured at 8 and 19 weeks, stratified by sex and diet. Error bars indicate standard deviation with *n* = 193–225 mice per group.
**Fig. S2.** Calcium is not strongly associated with diet, sex, or bone mass/density in diversity outbred mice. (A) Violin plot of calcium levels at 19 weeks across diets and sex. Sex and diet stratified scatter plots showing the relationship between calcium at 19 weeks and both (B) HDL Cholesterol and (C) non‐HDL Cholesterol. Sex and diet stratified scatter plots of the relationships between bone mineral content (D) and bone density (E) via DEXA scan and their relationships with cholesterol levels at 19 weeks. For (A), the *P*‐values represent the significance of diet and sex from a multivariate linear model. For (B–E), *P*‐values indicate the significance for the diet and sex‐adjusted relationship between cholesterol and the predictor from a multivariate linear model.

**Table S1.** Association between cholesterol at 19 weeks and other measured parameters. Spearman's correlation coefficients were calculated for each comparison with the number of mice (*n*), Spearman's Rho (estimate) and *P*‐value (cor.*P*.value) calculated for each term. Linear models were then constructed adjusting for both sex and diet for each comparison with cholesterol at 19 weeks. For this analysis, we report the beta coefficient (beta), standard error (SE), and *P*‐value (lm.*P*.value). Both sets of *P*‐values were adjusted for multiple comparisons by the method of Benjamini and Hochberg and reported as cor.*P*.adj and lm.*P*.adj respectively. Some parameters (albumin to creatine ratio, adiponectin at 8 weeks) were not measured for all groups, so only Spearman's Rho was estimated. Data are arranged in descending order by the absolute value of the correlation coefficient.

 

## Data Availability

The data that support the findings of this study are openly available in Github at https://github.com/BridgesLab/PrecisionNutrition and in Zenodo at https://doi.org/10.5281/zenodo.10408266 record number 10408266.
