# Supplementary figures and images for: Cross‐sectional association between blood cholesterol and calcium levels in genetically diverse strains of mice
Source: FEBS Open Bio. 2024 Jan 7;14(3):426–33. doi: 10.1002/2211-5463.13757 (PMC10909986; doi:10.1002/2211-5463.13757)

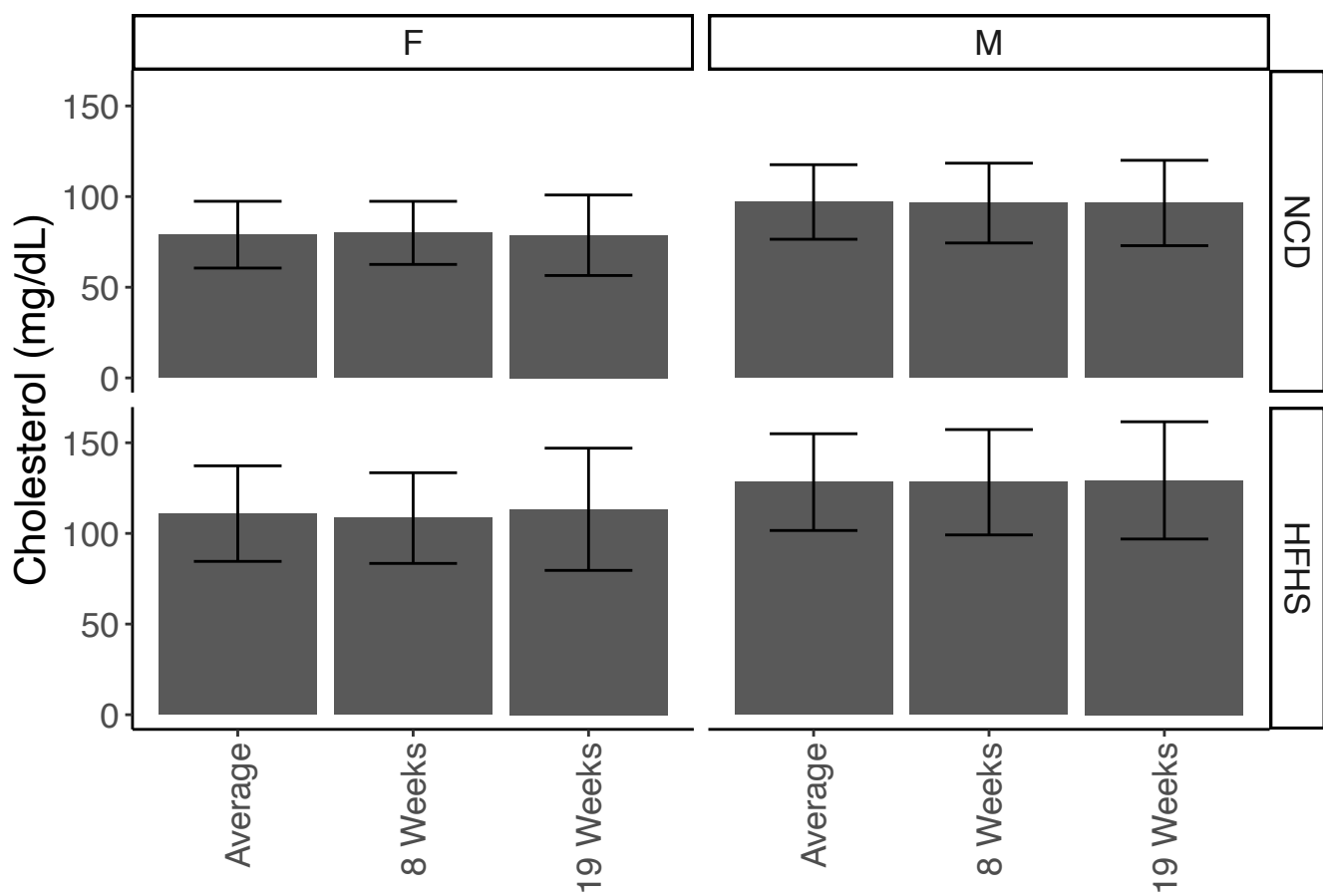

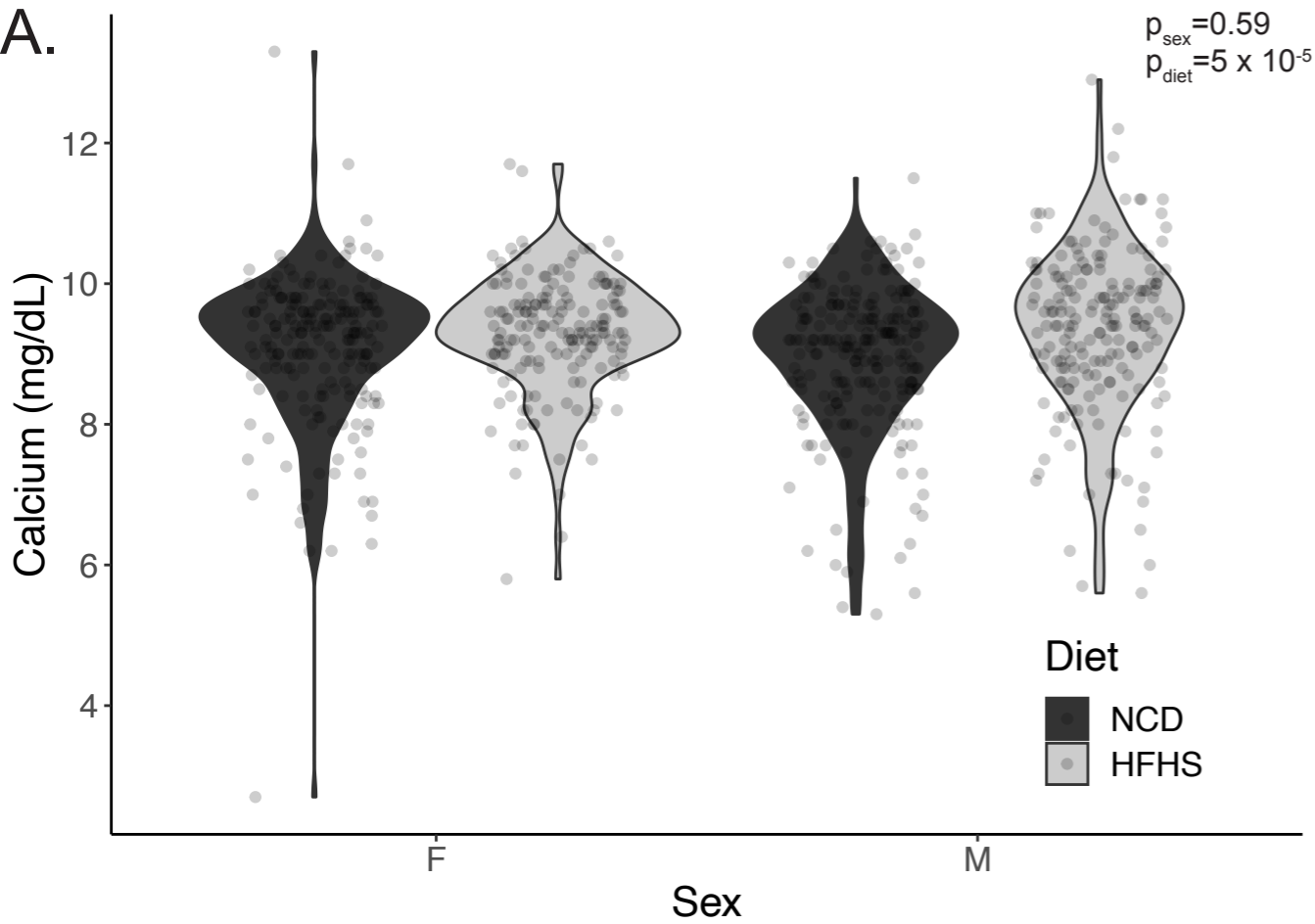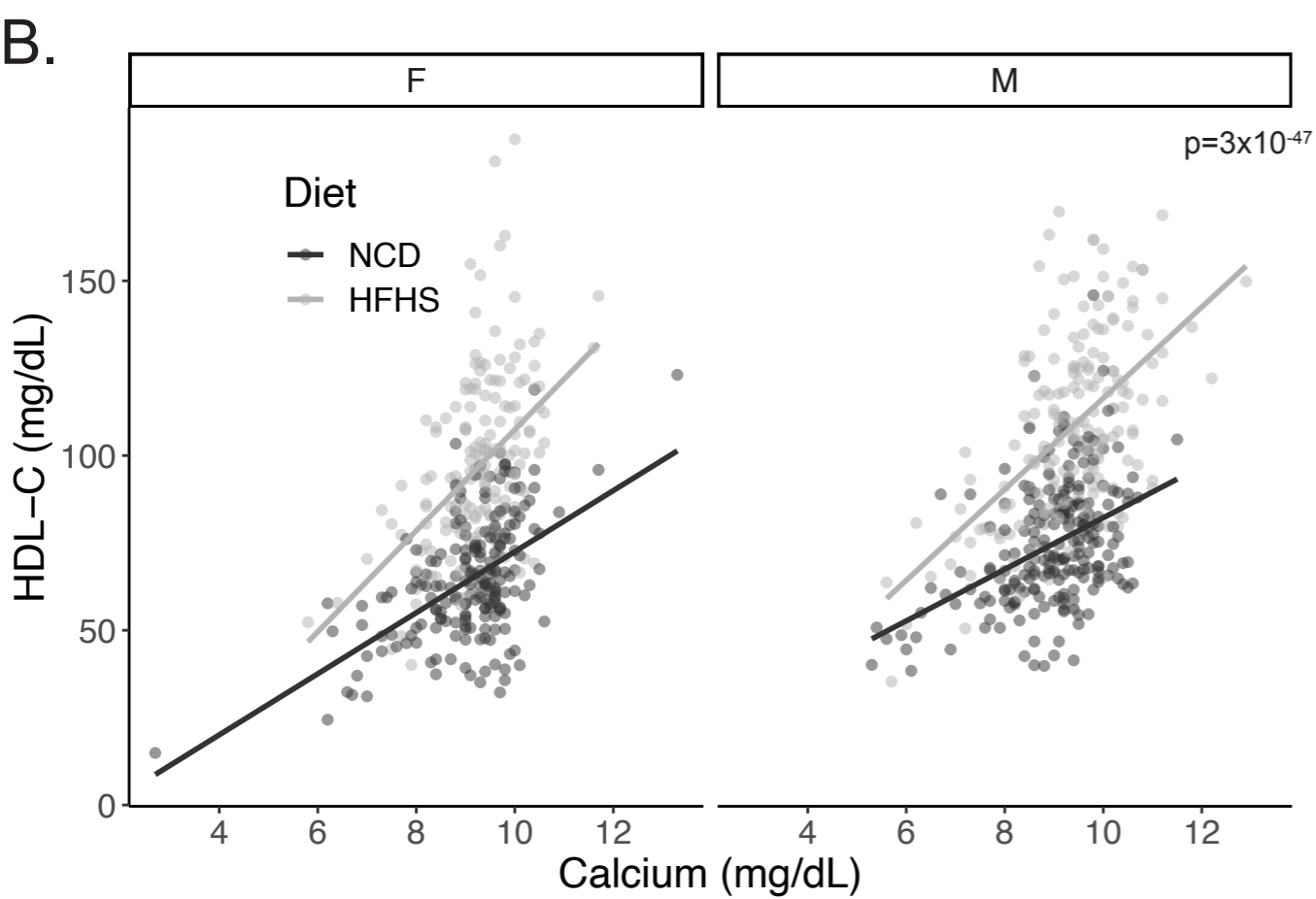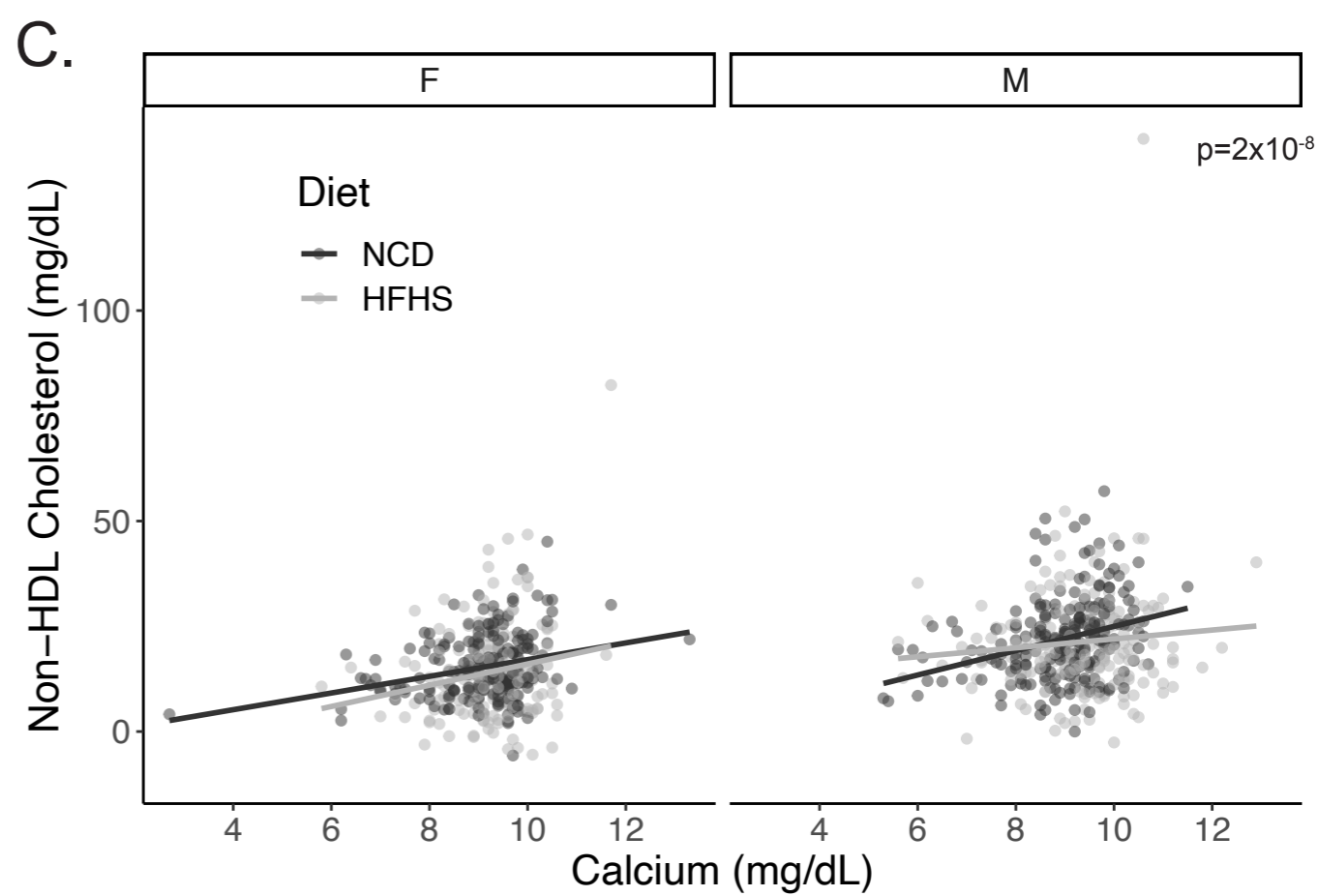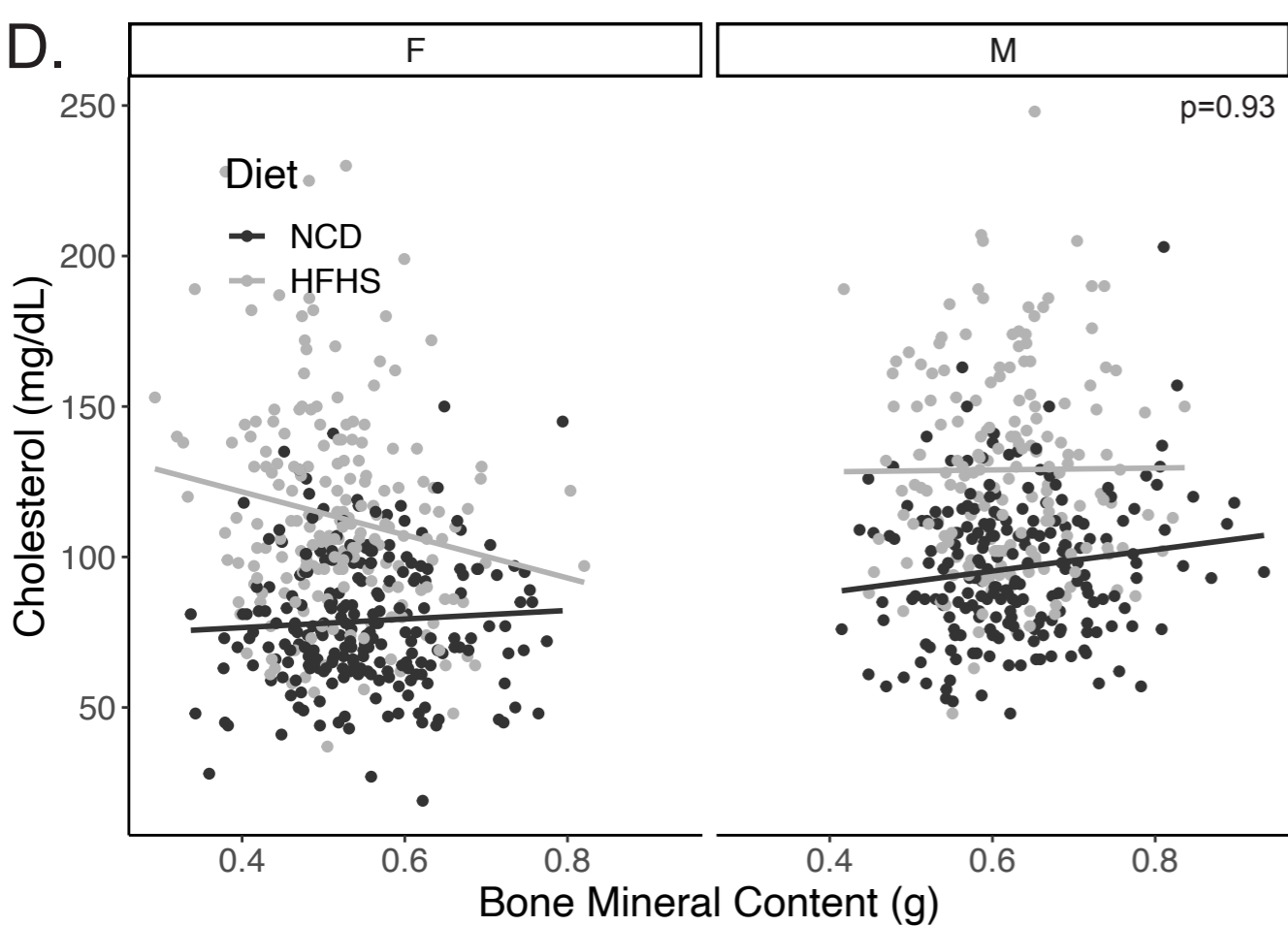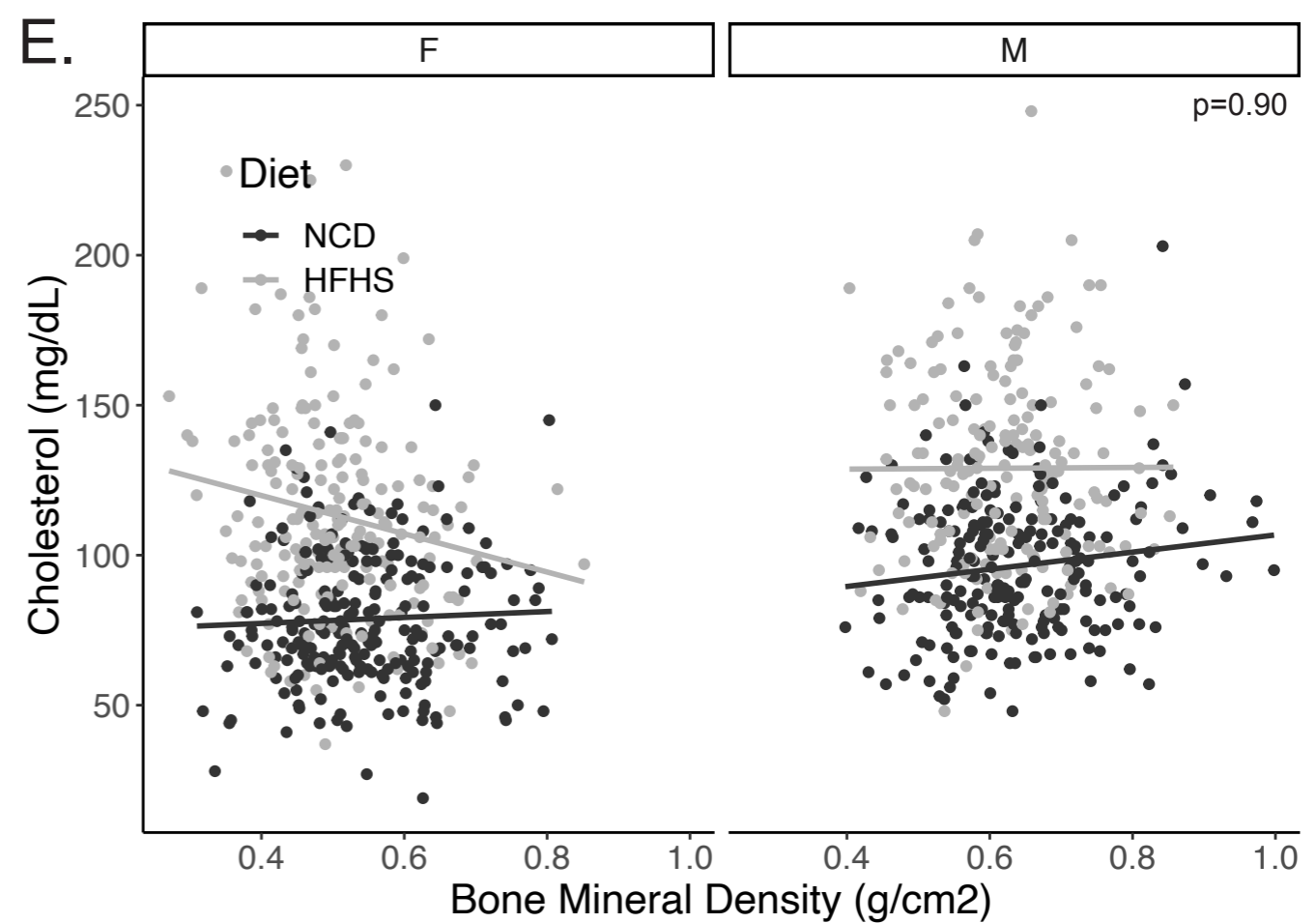

Supplement: Supplementary file 1 — Fig. S1. Cholesterol levels are stable across time in diversity outbred mice. Average cholesterol levels, and levels measured at 8 and 19 weeks, stratified by sex and diet. Error bars indicate standard deviation with n = 193–225 mice per group. Fig. S2. Calcium is not strongly associated with diet, sex, or bone mass/density in diversity outbred mice. (A) Violin plot of calcium levels at 19 weeks across diets and sex. Sex and diet stratified scatter plots showing the relationship between calcium at 19 weeks and both (B) HDL Cholesterol and (C) non‐HDL Cholesterol. Sex and diet stratified scatter plots of the relationships between bone mineral content (D) and bone density (E) via DEXA scan and their relationships with cholesterol levels at 19 weeks. For (A), the P‐values represent the significance of diet and sex from a multivariate linear model. For (B–E), P‐values indicate the significance for the diet and sex‐adjusted relationship between cholesterol and the predictor from a multivariate linear model. [file FEB4-14-426-s002.pdf]
