# Supplementary material for: Cross‐sectional association between blood cholesterol and calcium levels in genetically diverse strains of mice
Source: FEBS Open Bio. 2024 Jan 7;14(3):426–33. doi: 10.1002/2211-5463.13757 (PMC10909986; doi:10.1002/2211-5463.13757)
Supplement: Supplementary file 3 [file FEB4-14-426-s001.docx]

**Supplementary Figure 1: Cholesterol levels are stable across time in diversity outbred mice.** Average cholesterol levels, and levels measured at 8 and 19 weeks, stratified by sex and diet. Error bars indicate standard deviation with n=193-225 mice/group.

**Supplementary Figure 2: Calcium is not strongly associated with diet, sex or bone mass/density in diversity outbred mice.** A) Violin plot of calcium levels at 19 weeks across diets and sex. Sex and diet stratified scatter plots showing the relationship between calcium at 19 weeks and both (B) HDL Cholesterol and (C) non-HDL Cholesterol. Sex and diet stratified scatter plots of the relationships between bone mineral content (D) and bone density (E) via DEXA scan and their relationships with cholesterol levels at 19 weeks. For A, the p-values represent the significance of diet and sex from a multivariate linear model. For B-E, p-values indicate the significance for the diet and sex adjusted relationship between cholesterol and the predictor from a multivariate linear model.

**Supplementary Table S1: Association between cholesterol at 19 weeks and other measured parameters.** Spearmans’s correlation coefficients were calculated for each comparison with the number of mice (n), Spearman’s Rho (estimate) and p-value (cor.p.value) calculated for each term. Linear models were then constructed adjusting for both sex and diet for each comparison with cholesterol at 19 weeks. For this analysis we report the beta coefficient (beta), standard error (se), and p-value (lm.p.value). Both sets of p-values were adjusted for multiple comparisons by the method of Benjamini and Hochberg and reported as cor.p.adj and lm.p.adj respectively. Some parameters (albumin to creatine ratio, adiponectin at 8 weeks) were not measured for all groups, so only Spearman’s Rho was estimated. Data are arranged in descending order by the absolute value of the correlation coefficient.
